# Barcoding Genetically Distinct Plasmodium falciparum Strains for Comparative Assessment of Fitness and Antimalarial Drug Resistance

**DOI:** 10.1128/mbio.00937-22

**Published:** 2022-08-16

**Authors:** Manuela Carrasquilla, Ndey F. Drammeh, Mukul Rawat, Theo Sanderson, Zenon Zenonos, Julian C. Rayner, Marcus C. S. Lee

**Affiliations:** a Wellcome Sanger Institute, Wellcome Genome Campus, Hinxton, United Kingdom; b Max Planck Institute for Infection Biology, Berlin, Germany; c Medical Research Council Unit The Gambia at the London School of Hygiene and Tropical Medicine, Banjul, The Gambia; d The Francis Crick Institute, London, United Kingdom; e Biologics Engineering, Early Oncology, AstraZeneca, Cambridge, United Kingdom; f Cambridge Institute for Medical Research, University of Cambridge, Cambridge, United Kingdom; University of Geneva

**Keywords:** apicomplexan parasites, drug resistance, fitness, malaria

## Abstract

The repeated emergence of antimalarial drug resistance in Plasmodium falciparum, including to the current frontline antimalarial artemisinin, is a perennial problem for malaria control. Next-generation sequencing has greatly accelerated the identification of polymorphisms in resistance-associated genes but has also highlighted the need for more sensitive and accurate laboratory tools to profile current and future antimalarials and to quantify the impact of drug resistance acquisition on parasite fitness. The interplay of fitness and drug response is of fundamental importance in understanding why particular genetic backgrounds are better at driving the evolution of drug resistance in natural populations, but the impact of parasite fitness landscapes on the epidemiology of drug resistance has typically been laborious to accurately quantify in the lab, with assays being limited in accuracy and throughput. Here we present a scalable method to profile fitness and drug response of genetically distinct P. falciparum strains with well-described sensitivities to several antimalarials. We leverage CRISPR/Cas9 genome-editing and barcode sequencing to track unique barcodes integrated into a nonessential gene (*pfrh3*). We validate this approach in multiplex competitive growth assays of three strains with distinct geographical origins. Furthermore, we demonstrate that this method can be a powerful approach for tracking artemisinin response as it can identify an artemisinin resistant strain within a mix of multiple parasite lines, suggesting an approach for scaling the laborious ring-stage survival assay across libraries of barcoded parasite lines. Overall, we present a novel high-throughput method for multiplexed competitive growth assays to evaluate parasite fitness and drug response.

## INTRODUCTION

Antimalarial drug resistance repeatedly emerges first in certain regions of the world, a phenomenon that has been linked to particular genetic backgrounds that better tolerate resistance-associated polymorphisms (1–4). The independent origin of chloroquine and pyrimethamine resistance provided an example in which beneficial mutations to drug pressure were rapidly fixed in Southeast Asia and South America, before subsequent dissemination to sub-Saharan Africa (5–8). Even though these resistance mutations could have emerged locally in Africa, where both parasite diversity and burden is higher, a high fitness cost likely prevented them from being maintained: a rapid decrease in frequency of resistance alleles in Malawi as a consequence of chloroquine withdrawal supported the hypothesis that evolutionary forces were acting differently on these populations than in Southeast Asia and South America (9). Accurately and sensitively studying the impact of resistance-associated polymorphisms on both resistance and fitness is therefore key to defining the fitness landscapes of parasite populations globally.

The impact of drug resistance on parasite fitness has proven difficult to quantify in a laboratory setting. A major constraint on throughput is the fact that most fitness comparisons involve performing head-to-head competition assays with just two strains, a test line and a reference parasite, for which multiple readouts have been employed. One approach to detect the relative proportion of two alleles in a mixture is pyrosequencing, which uses the pyrophosphate released by polymerase incorporation of specific nucleotides to drive a luciferase-based enzymatic reaction. This light emission allows accurate quantification of the incorporation of nucleotides by using allele-specific primers. By this approach, the contribution of drug-resistant alleles can be determined by coculturing resistant and sensitive clones (2, 10). Another approach to head-to-head competitive assays is to use a non-fluorescent line of interest cocultured with a green fluorescent protein-competitor line, with flow cytometry reporting changes in relative proportion (11, 12). Quantitative PCR has also been employed to measure relative abundance of cocultured P. falciparum lines, by using probes detecting different alleles of drug resistance genes (13). These methods have highlighted the importance of measuring fitness in understanding the complexity of drug resistance in P. falciparum parasites, however none allows for simultaneous measurement of large numbers of lines.

The ability to track the growth of many cell lines in parallel has been an important tool for phenotyping in model organisms such as yeast (14) but is relatively new to apicomplexan parasites. Recent examples of this are the application of barcode sequencing (BarSeq) for high throughput growth phenotyping of gene essentiality in P. berghei, where systematic reverse genetics was used to generate knockout mutants and insert barcodes for fitness tracking during subsequent blood-stage growth (15). Gene essentiality has also been investigated in P. falciparum, where *piggyBac* transposon mutagenesis was used to generate pools of mutant parasites, and quantitative insertion site sequencing (QIseq) used to both identify insertion sites and track mutant growth (16). Another study by Sidik et al. (2016) used CRISPR/Cas9-based genome editing to target all genes across the genome of Toxoplasma gondii, with the unique gRNA for each gene acting as a barcode to quantify growth (17). All these studies measured the growth of particular knockout or insertion mutants within a pool by tracking unique short DNA tags by next-generation sequencing (NGS). Other approaches have also been developed to follow the growth of mutants within a pool, such as bulk segregant analysis (BSA), which measures fluctuations in allele frequencies in a mixed culture of progeny from *Plasmodium* genetic crosses (18). Amplicon sequencing of drug resistance alleles has also been used to follow changes in frequency of specific alleles in the presence or absence of drug selection (3). Given the severe public health challenge posed by antimalarial drug resistance, such pooled approaches provide an important opportunity to scale up parasite phenotyping and offer more scalable approaches to understand the fitness and drug sensitivity/resistance impact of natural or engineered genetic variation, and the influence of genetic background on buffering resistance driver mutations.

Here, we develop a barcode tagging approach for P. falciparum, which lacks the high transfection efficiencies of P. berghei or T. gondii and apply it specifically to the study of drug resistance and parasite fitness. We used CRISPR-editing to insert short barcode cassettes at a nonessential safe-harbor locus, the pseudogene *Pfrh3*, resulting in stable maintenance and segregation of a single-copy tag for each line. Critically, all barcodes were inserted at the same genomic site and flanked by the same sequences, meaning that, unlike amplicon sequencing of resistance alleles, multiple tagged lines could be pooled, grown together under different selective conditions, and their relative proportions quantified using a single PCR followed by next-generation sequencing. As proof-of-principle, we barcoded genetically and phenotypically diverse parasite strains from different geographic regions and with different drug resistance profiles. The barcoded lines were pooled to perform competitive assays to measure both inherent growth differences as well as drug response to antimalarial compounds. We also performed ring-stage survival assays (RSA) for artemisinin resistance using barcode sequencing as a readout, demonstrating the potential for complex assays to be carried out on laboratory strains, or the progeny of genetic crosses, using pooled high-throughput screening.

## RESULTS

### Generating a panel of uniquely barcoded parasite clones.

**FIG 1:**
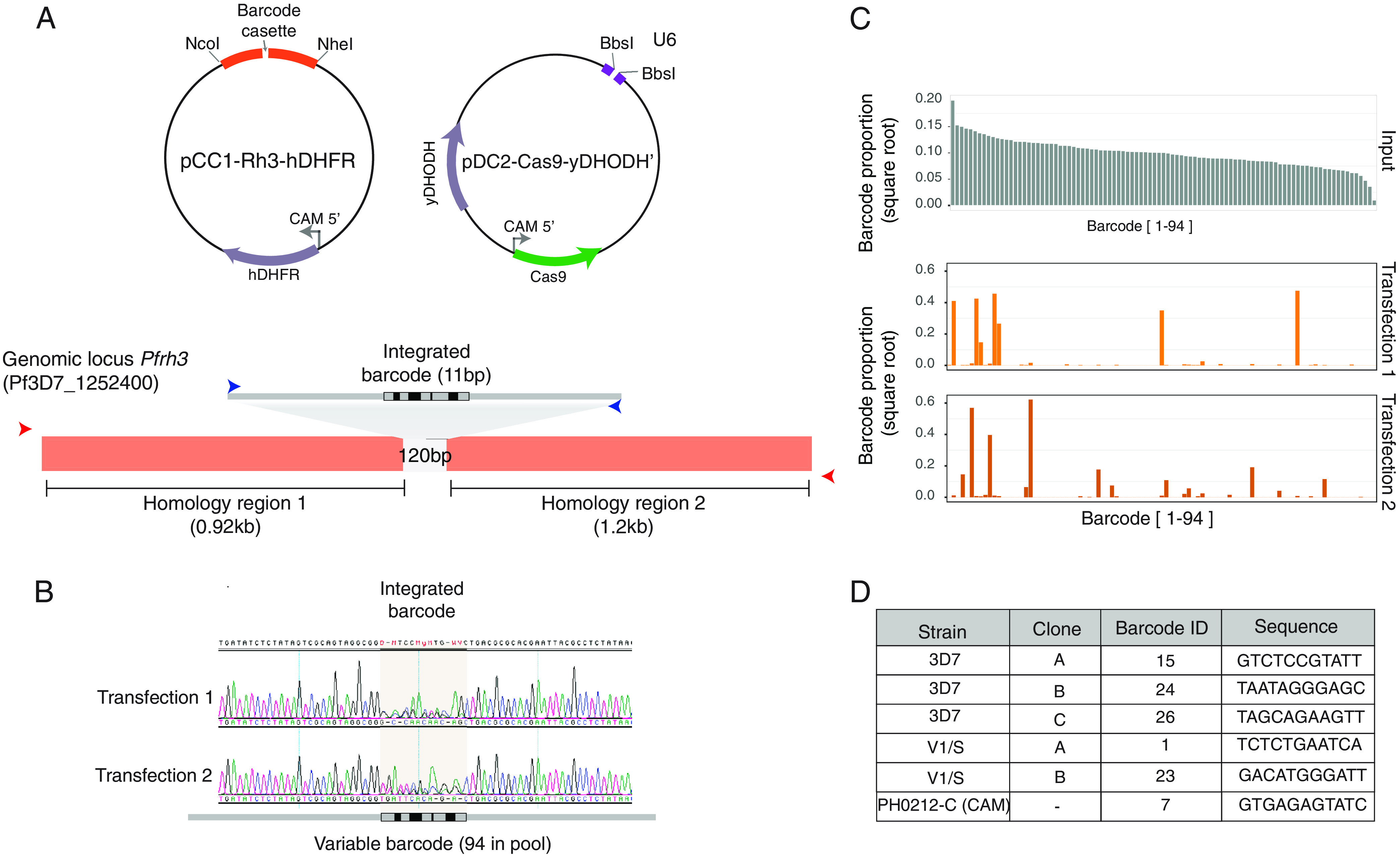
Editing of barcoded lines. (A) Graphical representation of the pCC1 donor plasmid and pDC2-Cas9 plasmid cotransfected for integration of a barcode cassette flanked by homology regions of *pfrh3* for homologous recombination. Red arrows in the bottom panel indicate the first PCR product generated from outside the homology regions to avoid episomal amplification. Blue arrows correspond to the second PCR inside the barcode cassette used for generating Illumina-compatible libraries. (B) Sanger sequencing chromatogram showing integrated barcodes after cotransfection of a pool of barcoded donor plasmids together with the pDC2-Cas9 vector. (C) Next-generation sequencing of the barcode region showing the input vector pool (top panel, gray) and the integrated barcodes, represented by the orange bars, for two independent transfections of 3D7. (D) Barcoded clones from the three strains used, the clone ID, the barcode ID from the pool of 94 barcodes, and the corresponding barcode sequence.

To develop a scalable approach for stable barcoding of different parasite lines, we generated a library of 94 different barcoded donor vectors encoding an 11 bp barcode sequence within a 120 bp cassette. This barcode library was assembled into a pCC1 vector with flanking ~1kb homology regions of the pseudogene *Pfrh3* (PF3D7_1252400) (19), resulting in a pool of up to 94 different donors, as recently described (20). To generate stable integrated barcodes, we generated an *rh3*-targeting Cas9/sgRNA expression plasmid, replacing the existing hDHFR cassette with the yeast y*DHODH* selectable marker to allow for coselection of Cas9-gRNA and donor plasmids (Fig. 1A) (21). The pool of barcoded donors was cotransfected with a Cas9/gRNA plasmid into three genetically diverse strains of P. falciparum*—*the reference 3D7 parasite, multidrug-resistant V1/S from Vietnam, and a recent Cambodian isolate (PH0212-C) harboring the C580Y mutation in *Pfkelch13* associated with artemisinin tolerance (Fig. 1D) (22). Primers targeting the flanking regions of the barcode insertion site within *rh3* were used to generate amplicons for barcode sequencing (BarSeq) using next-generation sequencing.

We first wanted to evaluate the complexity of barcode integration in a bulk culture of 3D7. Two independent transfections in this strain were initially assessed by Sanger sequencing, which revealed diverse nucleotide compositions at the barcode integration site within the bulk culture, consistent with multiple editing events having successfully taken place (Fig. 1B). BarSeq analysis of these transfections showed that 7–9 unique barcodes were recovered, confirming multiple different barcoded lines were present in the bulk population of transfectants (Fig. 1C). There was no strong bias toward barcodes that were more highly abundant in the original vector input pool (Fig. 1C, upper panel). Limiting dilution cloning was carried out to recover unique 3D7 barcoded lines. We then performed the same pooled transfections into strains CAM (Cambodia) and V1/S, observing a much lower complexity of the bulk population compared to 3D7, with only 1 and 2 barcodes recovered after cloning, respectively. Collectively, we selected six uniquely barcoded clones in total from these different genetic backgrounds (Fig. 1D) for use in downstream coculture experiments.

### Change in barcode proportion reveals growth phenotypes.

**FIG 2:**
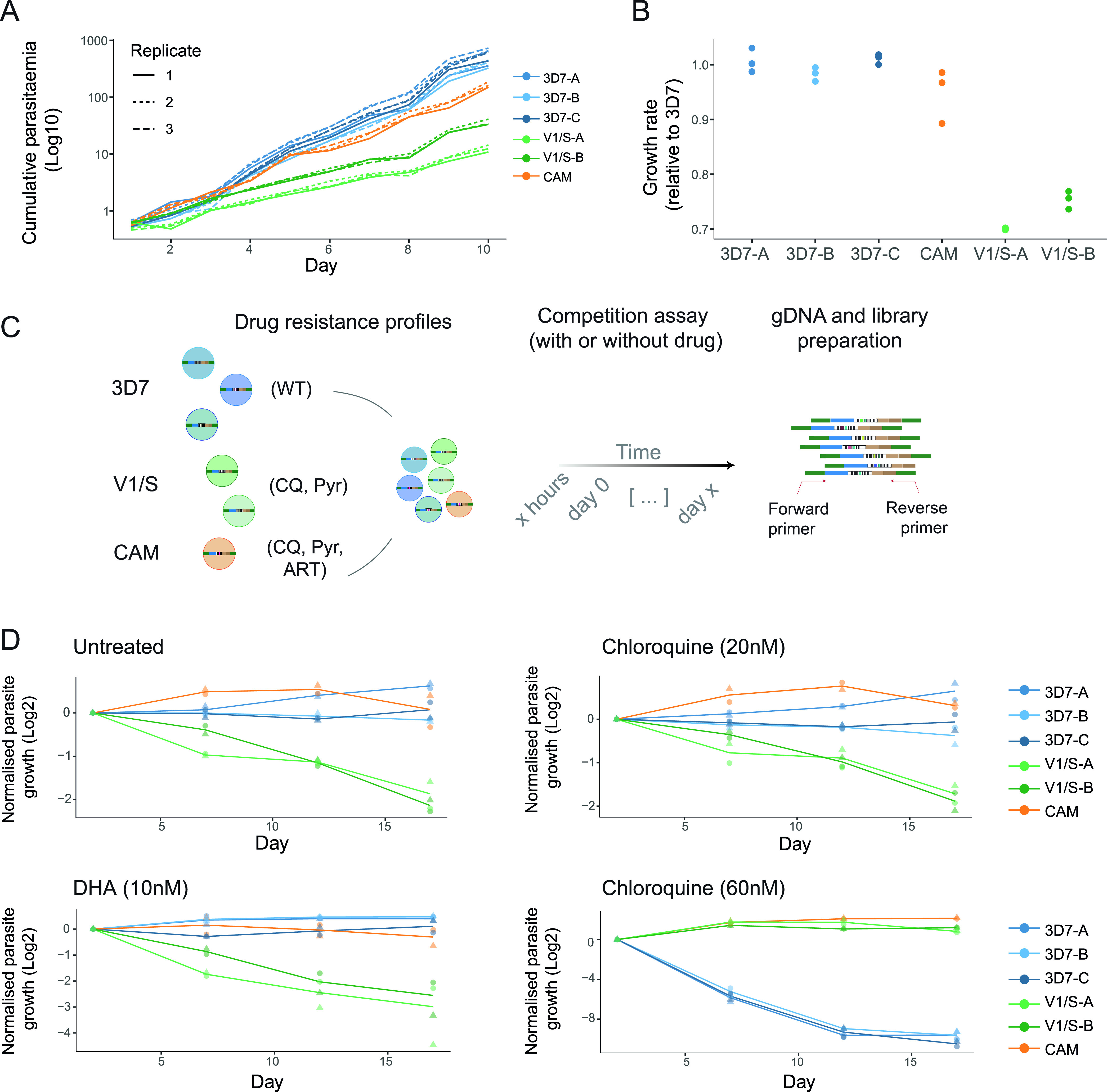
Competition assays to assess fitness and drug response. (A, B) Growth of barcoded strains was individually profiled in triplicate over 10 days. Their cumulative parasitemia over time was measured with flow cytometry (A) and normalized to the mean growth rate of the three 3D7 barcoded strains on each day (B, see methods section). (C) General experimental design for BarSeq. Genetically diverse strains with different drug sensitivity profiles (CQ-chloroquine, Pyr-pyrimethamine, ART-artemisinin - see methods) were pooled and grown together for multiple cycles in the presence or absence of drug selection. Samples were taken at multiple time points, genomic DNA extracted and nested PCRs performed to amplify the barcodes integrated into the *Pfrh3* locus of each strain. The relative proportion of each barcode was then quantified using NGS. (D) Competition assays lasting 18 days with two biological replicates (i.e., two independent pools of all six strains, grown in parallel). Following BarSeq, the relative proportion of each strain was normalized to its abundance at the first time point and log_2_-transformed (normalized parasite growth). Normalized growth of the pools in the absence of selection (untreated), or in the presence of constant chloroquine (20 nM and 60 nM) and DHA (10 nM) pressure.

Prior to starting competition experiments, we first wanted to determine the growth characteristics of each line individually. Each of the six barcoded lines were seeded in isolation at 0.5% parasitemia and their growth followed for 10 days using a standard flow cytometry approach to measure parasitemia. The 3D7 lines displayed the fastest growth followed closely by CAM, with the V1/S clones lagging more distantly (Fig. 2A and B).

**FIG 3:**
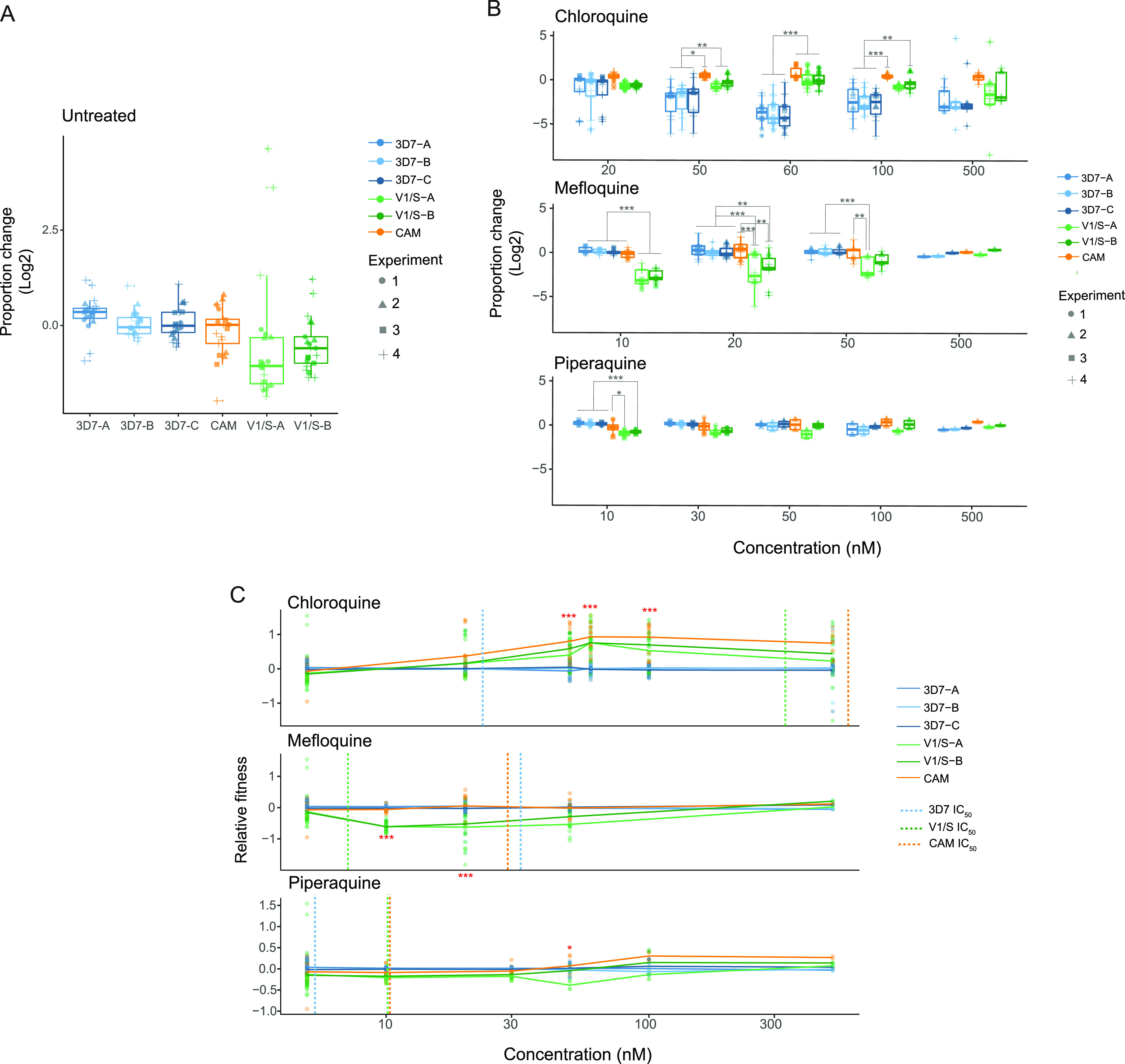
Drug response and relative fitness of barcoded strains (A–B) Competition assay of barcoded strains across four independent experiments (9 biological replicates). The ratio of the relative proportion of each strain at each time point (excluding the last day of the competition assay) with respect to the previous time point was log_2_-transformed (*y* axis). A one-way ANOVA with Tukey’s multiple-comparison test was performed between all strains and conditions, with significant differences shown (***, *P*-value < 0.001; **, *P*-value < 0.01; *, *P*-value < 0.05). Competition assay in the absence of drugs showed consistent growth disadvantage of two barcoded clones of V1/S, without reaching statistical significance (A). (B) Change in proportion of barcoded strains to determine their antimalarial response under three antimalarials: chloroquine (top), mefloquine (middle) and piperaquine (bottom) and at increasing concentrations starting from approximately the lowest IC_50_ of the three strains (Table S1 in the supplemental material). (C) Change in fitness relative to the control strain 3D7 including all biological replicates and log_2_-transformed. The *x* axis represents the concentration of chloroquine (top), mefloquine (middle) and piperaquine (bottom), with vertical lines indicating the IC_50_ of each strain coded by color (Table S1). Relative fitness of each line was normalized to the growth rate of the untreated control for the three independently barcoded lines 3D7 (3D7-A, 3D7-B, and 3D7-C). Significant differences relative to 3D7 were also determined at each concentration using a one-way ANOVA and Tukey’s multiple-comparison test between strains.

To compare these individual growth rates with a pooled competition approach, where the growth of each line can be measured by the change in relative abundance of barcodes, we mixed all six lines in equal proportion, in two independent pools (Fig. 2C) to compare their intrinsic growth phenotypes. Importantly, these competition assays included more than one uniquely barcoded clone for both the 3D7 and V1/S strains. These act as replicates for each other, allowing for internal biological controls to be compared simultaneously for these two strains. The independent pools were cultured for 18 days in the absence of antimalarials, with samples of genomic DNA being extracted at regular intervals for BarSeq (Fig. 2C). To generate growth curves derived from absolute read counts, we measured relative abundance of the different strains as the proportion of each barcode at a given time point. Overall, in the absence of drug pressure, the wild type 3D7 lines outcompeted the multidrug resistant V1/S lines but not the CAM parasite (Fig. 2D and 3A), consistent with their individual growth phenotypes. Notably, the three replicates of the 3D7 lines within the pool were internally consistent and showed no appreciable difference in growth between them, as expected (Fig. 2D).

10.1128/mbio.00937-22.4 TABLE S1 IC_50_ data for 3D7, V1/S and CAM. Download Table S1, XLSX file, 0.01 MB. Copyright © 2022 Carrasquilla et al. 2022 Carrasquilla et al. https://creativecommons.org/licenses/by/4.0/ This content is distributed under the terms of the Creative Commons Attribution 4.0 International license.

We explored the potential impact of fluctuations in seeding proportion on outcomes. Despite aiming to seed each line at equal proportion, actual proportions at the start of each assay varied (Fig. S1A in the supplemental material). Nonetheless, behavior of individual lines, as well as collectively for the three strains when data was aggregated across strain background yielded similar outcomes regardless of seeding proportion, suggesting they represent the intrinsic growth of each strain (Fig. 3A and Fig. S1B and C).

10.1128/mbio.00937-22.1 FIG S1 Strain proportions for BarSeq experiments. Starting proportions for the four different experiments were measured and compared with the expected proportions across timepoints (TP), excluding the last timepoint for analysis due to variability from low V1/S counts. (A) Relative proportion of each strain over time. (B) The ratio between the last (TP3 or 4) and first timepoints was log2-transformed (y axis) and individually barcoded strains and replicates were aggregated, showing an overall consistent behavior in growth regardless of observed starting proportion. (C) Starting proportion aggregated per strain over time and a linear regression (gray line), shows that consistently 3D7 increases over time, whereas V1/S gets outcompeted (R of -1 for experiments 1-3 and -0.4 for experiment 4). Dashed lines represent the expected seeding proportions for each strain aggregated. Download FIG S1, EPS file, 0.8 MB. Copyright © 2022 Carrasquilla et al. 2022 Carrasquilla et al. https://creativecommons.org/licenses/by/4.0/ This content is distributed under the terms of the Creative Commons Attribution 4.0 International license.

### Relative drug sensitivity of strains measured by BarSeq.

We next asked whether BarSeq could capture changes in growth rates in the presence of antimalarial drugs, detecting changes in sensitivity at increasing drug pressure. Drug concentrations were based on the IC_50_ of the 3D7 sensitive strain (Table S1 in the supplemental material). Both the V1/S and CAM strains have the chloroquine-resistant allele of *pfcrt*, and as chloroquine pressure was increased from approximately 1×IC_50_ to 3×IC_50_ of the sensitive strain, the growth advantage of 3D7 was reversed and both CAM and V1/S rapidly dominated the parasite population (Fig. 2D).

We further expanded our analysis to other clinically used antimalarials, dihydroartemisinin (described below), mefloquine and piperaquine. Mefloquine has been used in combination with artesunate since significant resistance in Thailand and Cambodia emerged following its use as a monotherapy (23, 24). However, recent and rapid propagation of resistance to both partner drugs has rendered the use of some ACTs completely ineffective in some areas of Southeast Asia (25, 26).

When the pooled strains were selected using mefloquine at 10 nM, a concentration below the IC_50_ for 3D7 (32 nM), we observed a significant hypersensitivity phenotype for V1/S (Fig. 3B and Fig. S2A and B in the supplemental material), consistent with our experimental values obtained from standard 72h dose-response assays (Table S1) as well as previous findings (27). Thus, mefloquine exposure inverts the 3D7-V1/S fitness relationship observed under chloroquine pressure (Fig. S2A and B). At low levels of piperaquine (10–30 nM), no clear change in overall profile was observed compared with untreated, with V1/S being gradually outcompeted, presumably due to its inherently lower growth rate (Fig. 2B) rather than drug response. Higher piperaquine concentrations appeared to provide a modest but not significant growth advantage for the CAM line, and the inherent growth disadvantage of V1/S was also partially negated under piperaquine pressure (Fig. 3B and Fig. S2B). These results are consistent with the 2-fold higher IC_50_ for piperaquine of both the CAM and V1/S lines relative to 3D7 (Table S1) and support an overall advantage of the CAM strain at higher piperaquine concentrations attributable to higher fitness relative to V1/S and higher IC_50_ relative to 3D7. Treatment at the highest concentrations of mefloquine and piperaquine (500 nM) resulted in killing of all lines (Fig. 3B and Fig. S1B) as this concentration was well in excess of 10×IC_50_ in each case. In contrast, 500 nM chloroquine was less than 2×IC_50_ for V1/S and slightly below the IC_50_ for CAM, and thus still yielded meaningful data due to ongoing parasite proliferation (Fig. S2A and B).

10.1128/mbio.00937-22.2 FIG S2 Drug response of barcoded strains at different concentrations in two independent experiments. (A) Normalized growth (y axis) was calculated as the ratio of each proportion at each timepoint to the starting proportion for the untreated condition and at increasing concentrations of chloroquine (left) starting at the IC_50_ for 3D7 (20 nM), 50, 100 and 500 nM over 17 days. Right panel shows normalized growth in presence of mefloquine at concentrations ranging from 10 nM (IC_50_ for V1/S and CAM), 20 and 50 nM over 17 days or lasting 8 days at the highest concentration (500 nM). (B) Normalized growth (y axis) in the untreated control and increasing concentrations of chloroquine, mefloquine and piperaquine. For chloroquine (left), 50 and 100 nM were used over a period of 8 days, for mefloquine (middle) 20 and 50 nM over 8 days and piperaquine 50 and 100 nM for 8 days (right). 500 nM treatments for all three antimalarial drugs were performed for 6 days. Download FIG S2, EPS file, 1.5 MB. Copyright © 2022 Carrasquilla et al. 2022 Carrasquilla et al. https://creativecommons.org/licenses/by/4.0/ This content is distributed under the terms of the Creative Commons Attribution 4.0 International license.

We then calculated the relative growth per day for each barcode at each time point, normalizing each data point to the mean value from the three individually barcoded clones of 3D7. By calculating the mean to all the individual barcoded replicates measured, we were able to establish fitness relationships between strains. These relationships were more easily visualized by plotting the change in fitness relative to 3D7 over different drug concentrations and allowed us to statistically compare strains at a given concentration (Fig. 3C). Overall, these results illustrate the capacity of the BarSeq assay to capture how relative fitness is modulated by drug sensitivity, and the potential of the assay to test multiple drugs and drug concentrations in parallel.

### Artemisinin response measured by barcode sequencing.

There is a pressing need to more fully understand how parasite populations, particularly in Southeast Asia, are adapting to the frontline antimalarial artemisinin. In addition to distinct mutations in the *pfkelch13* gene, of which the most common is C580Y, there is also evidence that the genetic background is instrumental in buffering potential fitness costs of different *pfkelch13* alleles (2, 3). Efforts to dissect the contribution of the genetic background in compensating for the acquisition of artemisinin-resistance are now being aided by the use of genome editing to insert *pfkelch13* alleles into distinct strains, and genetic crosses using the humanized mouse model (18, 28). However a major bottleneck is the subsequent large-scale phenotyping of the resulting lines, both in terms of fitness cost incurred in the absence of drug and individual artemisinin tolerance, which is conventionally measured using the laborious ring-stage assay (RSA) method (29). We took advantage of our pool containing a Cambodian isolate with the PfKelch13-C580Y allele to explore whether the multiplex barcode approach could be exploited to phenotype artemisinin sensitivity.

Continuous exposure, without regular synchronization and across the entire intraerythrocytic cycle of the parasite pool to 10 nM dihydroartemisinin (DHA), the active metabolite form of artemisinin, using similar conditions to the experiments above did not yield significant differences between strains (Fig. 2D). Artemisinin has a short half-life, which combined with the frequency of treatment failure (30), has resulted in the need to develop assays to accurately measure treatment failure due to resistance. Standard inhibitory assays have failed to do so, as they do not show a significant shift in the resistant lines that is well correlated with the slow-clearance phenotype in clinical cases. As an alternative, the RSA involves treatment of early rings, a stage shown to be resilient in slow-clearing clinical isolates, at a high concentration of DHA (29). We first tested a simplified and less laborious approach, performing daily 6 h pulses of 10 nM DHA on unsynchronized cultures without consideration of stage, over a period of 17 days. However, no enrichment of the Kelch13-mutant strain was observed (Fig. S3A–C in the supplemental material).

**FIG 4:**
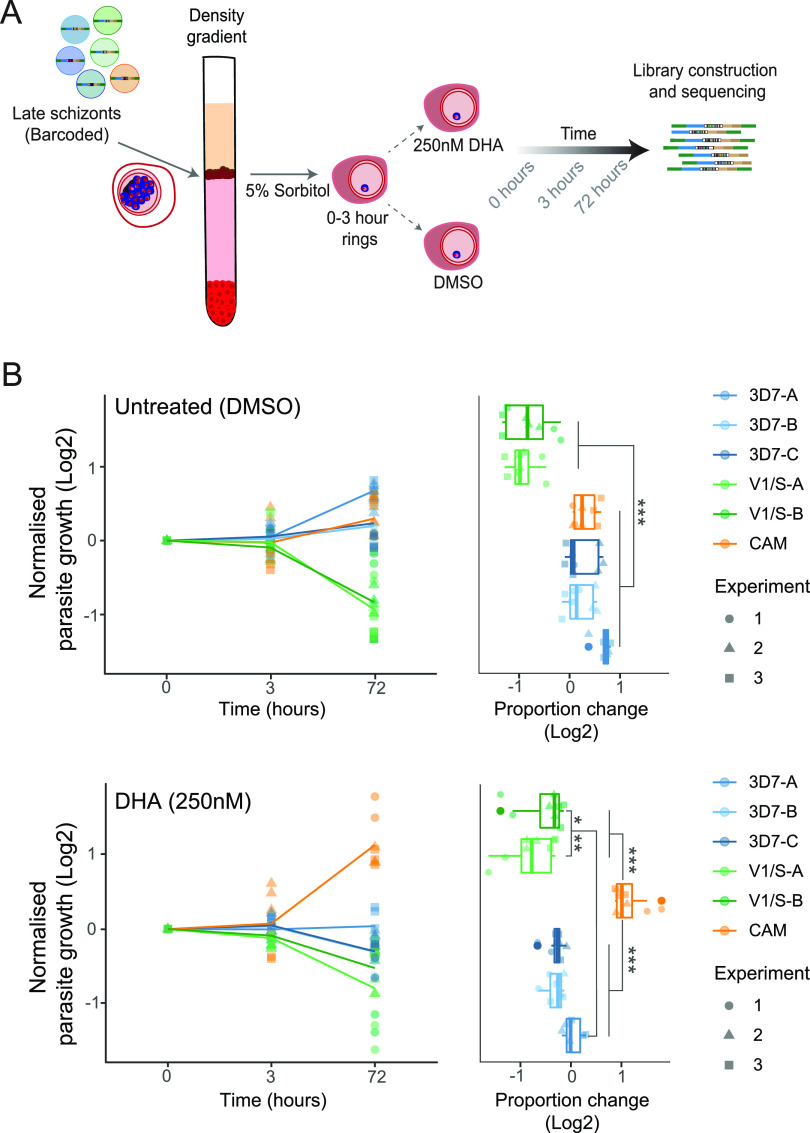
Barcode sequencing to assess artemisinin response of strains. (A) Experimental design for RSA-like experiment to assess artemisinin sensitivity. Barcoded strains were individually grown until the schizont stage and combined at equal ratios followed by a density gradient (63% Percoll) enrichment. Next, 0–3 h rings were synchronized with 5% Sorbitol and split in different replicates for the two conditions: Untreated (DMSO) and DHA (250 nM). Genomic DNA was collected at 0, 3 and 72 h and BarSeq libraries were generated as previously described. (B) RSA-like experiment showing growth of strains over the 72-h assay. *y* axis shows the log_2_ of each barcode proportion normalized by the first time point (0 h), followed by 3 and 72 h (left panel) for three independent experiments. Right panel shows the change in proportion from 0 to 72 h. Statistical significance between strains was calculated with one-way ANOVA and TukeyHSD (***, *P*-value < 0.001; **, *P*-value < 0.01; *, *P*-value < 0.05).

10.1128/mbio.00937-22.3 FIG S3 Competition assay with DHA using daily pulsing with 10 nM for 6 h. To complement Fig. 2D and 4. (A,B). DHA at 10 nM was pulsed for 6 h every 24 h in two independent experiments (four biological replicates) to mimic the short half-life of the drug. Normalized growth for each strain was calculated as the ratio of each proportion to the starting proportion and subsequently log2-transformed (see methods). Note that panel A relates to the experiment shown in Fig. 2D. (C) The change in proportion was calculated as the ratio of the relative proportion of each strain at each timepoint (excluding the last day of the competition assay) with respect to the previous timepoint and log2-transformed (y axis). A one-way ANOVA with Tukey’s multiple comparisons test was performed between all strains and conditions, and significant differences are shown (***, *P*-value < 0.001; **, *P*-value < 0.01; *, *P*-value < 0.05). Download FIG S3, EPS file, 1.1 MB. Copyright © 2022 Carrasquilla et al. 2022 Carrasquilla et al. https://creativecommons.org/licenses/by/4.0/ This content is distributed under the terms of the Creative Commons Attribution 4.0 International license.

We next adopted a modified version of the RSA using a 6 h pulse treatment but lower DHA concentration (250 nM as opposed to 700 nM; Fig. 4A) to increase the relative survival of all strains and capture measurable barcodes, as the resistant strain forms only a small proportion of the total parasite pool. Using this experimental setup, BarSeq now revealed the expected response of the different strains to the drug, with CAM being the only line with increased survival, in concordance with the presence of the Kelch13-C580Y mutation and with those validated by other groups (2, 22, 31, 32) (Fig. 4C). Overall, this assay shows an approach for robust simultaneous phenotypic assessment of the drug response of multiple strains in a pool to the frontline antimalarial artemisinin and offers the promise of ultimately screening hundreds of barcoded isolates on a single lane of Illumina MiSeq.

## DISCUSSION

To accelerate efforts toward malaria elimination, novel antimalarials will be required to counter the current wave of multidrug resistant parasites rapidly emerging. Having tools to better understand drug mode of action will be essential as we evaluate how new compounds function with current antimalarials, with a view to future combination therapies (33, 34). The complex interplay of drug resistance mutation acquisition and its overall impact on fitness has been highlighted in several studies (3, 18, 35–37). However, being able to experimentally observe how drug-exposure interacts with particular genotypes to modulate overall parasite fitness has been challenging and will better anticipate how these will perform in natural populations and polyclonal infections. In this work we present a method that could take us in that direction by tracking the competitive advantage of multiple P. falciparum strains, each carrying unique genomic barcodes, over time and drug exposure levels.

The use of BarSeq in P. berghei parasites using the *Plasmo*GEM vector resource has allowed functional profiling of gene knockouts at genome-scale (15). As a complementary approach, we evaluated whether a similar approach could be applied to P. falciparum for understanding the fitness effects of drug resistance mutations *in vitro*, and their relevance in the current epidemiology of drug resistance. Unlike the more facile genetic manipulation of P. berghei, to do this in P. falciparum we used CRISPR-based editing to generate tagged lines that were uniquely barcoded at a constant safe-harbor genomic region, the pseudogene *rh3*. We transfected CRISPR-editing donors with 94 different barcodes within a single bulk transfection and subsequently obtained individually barcoded parasites via dilution cloning. Due to the low transfection efficiency of P. falciparum in general and field isolates, in particular, we only recovered a limited number of barcoded clones using this approach. Scaling barcoding to larger numbers of parasites will require more efficient strategies, including arrayed transfection of individual barcodes (e.g., in 96-well plates) (16) rather than the pooled transfection used here, replicate transfections for poorly transfectable strains such as recently adapted field isolates, identification of highly efficient gRNAs to improve editing and positive-selection of integrants to obviate cloning (38).

Nonetheless, we generated sufficient clones to assay multiple strains in parallel, with replicates for two strains, and measured *in vitro* growth under a range of selection conditions. The sensitivity and throughput of the approach allowed us to observe that in the absence of drug pressure the more recent Cambodian isolate was unexpectedly nearly equivalently fit to 3D7, which has been cultured for decades, whereas V1/S had a distinct fitness disadvantage to both strains. The influence of genetic background to sustain fitness in the presence of K13 mutations has been explored by genome editing; in one study, Straimer et al. (2) observed that the K13-C580Y mutation in V1/S had a strong fitness cost whereas a Cambodian isolate (distinct from the CAM strain in our study) retained fitness. Application of increasing chloroquine pressure reversed the V1/S disadvantage, consistent with the presence of the mutant *pfcrt* allele, whereas mefloquine pressure restored the advantage of 3D7 over V1/S. Notably the CAM strain remained competitive under all conditions. These experiments confirm the impact of known drug resistance-associated mutations, as well as visualize inflection points where drug susceptibility and parasite fitness intersect.

Implementing this CRISPR-tagging and barcode sequencing approach to dissect fitness-conferring factors in the evolution to the frontline antimalarial artemisinin could have enormous value given the current wave of artemisinin resistant parasites that have emerged independently in different geographical locations (31, 39, 40), and with questions remaining as to whether parasites in other regions of the world harbor a genetic background that might favor the emergence and spread of these resistance-associated mutations. Constant exposure to DHA or pulsing of unsynchronized cultures was insufficient to enrich the Kelch13-mutant CAM strain. However, by performing an RSA-like drug exposure for the pooled parasites in which tightly-synchronized early rings were pulsed with high concentration of DHA for 6 h to mimic the RSA protocol, we were able to detect enrichment of the Kelch13-mutant CAM parasite. Thus, this approach could potentially be scaled to tens or hundreds of barcoded strains that could be profiled simultaneously unlike the standard RSA approach where each strain is assayed separately. One potential challenge would be the necessity to obtain tightly synchronized cultures, with the risk that synchronization of a pool would result in loss of some lines. However, this could potentially be mitigated by using different synchronization schedules for replicate pools.

Several recent studies have developed genetic crosses of P. falciparum isolates using humanized mouse models (28), aimed at understanding drug resistance determinants and how genetic background supports the resistance phenotype. Achieving this will require phenotyping of potentially hundreds of clonal progeny of resistant and sensitive parental strains, and changes in both drug sensitivity as well as in fitness measured by *in vitro* growth. One powerful method for identifying causal determinants in this context is bulk segregant analysis (BSA), which also operates using pooled parasites (28). Whereas the BarSeq approach described here uses the barcode as a proxy for parasite genotype, BSA measures the allele frequency directly. Notably, a recent study performed BSA on a pool of progeny that had undergone synchronization and an artemisinin pulse (41). Similar to our observations, their study found that only the ring-treated synchronized cultures yielded Kelch13-mutant signals, reflecting the stage when protection is afforded by mutant Kelch13. Brenneman et al. (41) also performed a titration of DHA to identify pulse concentrations that allowed partial survival of nonresistant lines, identifying 50 and 100 nM as optimal, similar to the 250 nM concentration used here. Although the BSA approach bypasses the up-front labor of barcode tagging the progeny, the generation of barcoded progeny provides some specific advantages in dissecting more complex polygenic traits by preserving haplotype information, while still allowing bulk analysis using assays such as competitive fitness and the artemisinin RSA to be tested simultaneously on large pools. Barcoding would also be complementary to traditional quantitative trait loci mapping approaches, as whole genome sequencing would allow barcode enrichment to be correlated with pre-established genotypes to decipher alleles under selection. In addition, barcoding progeny would guard against potential cross-contamination, allowing rapid identification of lines without whole genome sequencing.

Barcode tagging could also be used to track mutants generated by conventional knockout methods or random mutagenesis such as *piggyBac* transposon insertion. Competitive growth of thousands of *piggyBac* mutants generated by pooled transfections was successfully measured using quantitative insertion-site sequencing (QIseq) (16). However, barcoding of individual *piggyBac* lines could be a complementary approach that allows simplified amplification of a constant barcode cassette rather than the more complex QIseq protocol that requires fragmentation of genomic DNA, adapter ligation and sequencing of both 5′ and 3′ insertion sites (42).

Studies in other systems highlight the potential resolution achievable by barcode sequencing. The PlasmoGEM P. berghei KO screen tracked pools of ~100 barcoded lines to measure relative fitness (15). At a much larger scale, studies using the yeast model Saccharomyces cerevisiae have demonstrated that up to 500,000 clonal lineages could be tracked over hundreds of generations, revealing the impact of accumulating mutations on shaping fitness landscapes (14). Similarly, our relatively short-term assays were sufficient to reveal strain differences, particularly in the context of drug pressure, but extended time frames may be required to discern more subtle fitness differences such as between genetic cross progeny.

Recent years have seen an increase in the scale of adaptation of field isolates to culture (43, 44), and generating diversity via *in vitro* evolution of resistance to chemically diverse antimalarial compounds (45). By barcoding and then assaying parasites encompassing a wide range of genomic diversity available in the field we should be able to understand how resistance mutations, compensatory changes, and genetic background interweave to shape the fitness landscape. Future studies will further expand the scope of tracking barcoded parasites to include other stages of the life cycle (e.g., transmission stages), as well as measuring growth under alternative host environments that will ultimately increase our understanding of biological fitness in P. falciparum. One intriguing example would be to uniquely barcode multiple representatives of a single recently adapted isolate. Exposure of each clone to different culture times or regimens, followed by competitive growth assays would allow potentially subtle growth adaptations to be tracked and traced back to specific clones. Another compelling application would be the creation of a library of barcoded parasites that collectively represent all the major drug targets and resistance mechanisms currently known. This would provide a single pooled resource that could be used to comprehensively determine the fitness liabilities of different resistance pathways, and rapidly interrogate new antimalarial compounds to identify and potentially eliminate those with known modes-of-action.

## MATERIALS AND METHODS

### Parasite cultures.

Parasite strains used throughout this work were routinely cultured and propagated in human red blood cells provided by anonymous donors from the National Health Services Blood and Transplant (NHSBT). Informed consent was obtained by NHSBT as part of their recruitment process, and the use of RBCs was in accordance with relevant guidelines and regulations, with approval from the NHS Cambridgeshire Research Ethics Committee and the Wellcome Sanger Institute Human Materials and Data Management Committee. Culture media used throughout this work was as previously described (46), with a gas mixture of 1% O_2_, 3% CO_2_, and 96% N_2_, and maintained at 37°C. Synchronization of cultures was performed using 5% (wt/vol) sorbitol in water as previously described (47) or with a Percoll gradient as previously described (48). Parasite strains 3D7 (a clone of N54 from 19870) (49) and V1/S (a clone of the Vietnam V1 isolate from 1990) (50) were obtained from MR4, and PH0212-C (CAM) was isolated in 2010 by Rick Fairhurst in Pursat, Cambodia (22). As summarized in Fig. 2C, both the V1/S and CAM strains have the chloroquine-resistant allele of *pfcrt* and mutant *dhfr* that confers resistance to antifolate drugs. The CAM strain has mutant *pfcrt* and *dhfr*, as well as the K13-C580Y mutation that affords tolerance to artemisinin.

### Microscopy.

Microscopy was performed with standard blood smears to determine parasitemia. A small aliquot of culture was smeared on a glass slide, fixed with 100% methanol and stained with a 10% Giemsa solution (Sigma-Aldrich). 10 fields of ~100 RBCs were counted.

### Cloning of parasites by limiting dilution.

Parasites recovered after transfection were cloned using a limiting dilution protocol modified from (51). Parasites were diluted into a 96-well plate at 0.5–0.8 parasites per well at 1.8% hematocrit, for a recovery of 50% of the plate corresponding to clonal parasites. For rapid screening of positive wells, a DNA stain SYBR green method was used, and both empty wells or those with much higher fluorescence than the average (likely with more than one clone) were discarded.

### Generation of barcoding vectors.

The barcoding approach was adapted for P. falciparum from PlasmoGEM (52). We recently described successful transfection of episomally-maintained pools of barcoded vectors (19). Here we used these pools together with CRISPR/Cas9-expressing vectors for barcode integration into *Rh3* (PF3D7_1252400). In brief, the barcode-containing region from 96 different pGEM bacterial stocks was amplified simultaneously after individually growing and pooling them (TB medium with 30 μg/mL of kanamycin). Primers p212 and p219 (Table S4 in the supplemental material) were designed to amplify the 120 bp barcode amplicon with overlap sequences to a NheI/NcoI digested P. falciparum vector pCC1 (19). Gibson cloning (53) was used for integration of amplicon regions in-between two homology regions of *Pfrh3* (Fig. 1A), each spanning approximately 1 kb.

10.1128/mbio.00937-22.6 TABLE S3 Raw data for artemisinin RSA assays (BarSeq). Download Table S3, XLSX file, 0.02 MB. Copyright © 2022 Carrasquilla et al. 2022 Carrasquilla et al. https://creativecommons.org/licenses/by/4.0/ This content is distributed under the terms of the Creative Commons Attribution 4.0 International license.

10.1128/mbio.00937-22.7 TABLE S4 List of primers. Download Table S4, XLSX file, 0.01 MB. Copyright © 2022 Carrasquilla et al. 2022 Carrasquilla et al. https://creativecommons.org/licenses/by/4.0/ This content is distributed under the terms of the Creative Commons Attribution 4.0 International license.

CRISPR/Cas9 plasmids were generated using a pDC2 vector backbone (54), expressing Cas9 under a P. falciparum-specific promoter and a single guide RNA under a U6 promoter for RNA Pol III. We replaced the hDHFR positive selection cassette for yDHOH (55) to select for cotransfected plasmids.

### Parasite transfections.

Transfections were performed using the pool of 94 barcoded vectors as well as the CRISPR/Cas9-expressing vectors on ring-stages at 5–10% parasitemia. A Lonza Nucleofactor 4D was used with the program P3-CM150. Plasmid DNA composed of a 1:1 mixture of the targeting and the donor vectors was resuspended in 100 μL of P3 buffer (Lonza), containing 3 μL ATP (625 mM). Parasites were centrifuged and the DNA solution was used to resuspend 100 μL of packed RBCs. Selection with WR99210 was applied 24 h posttransfection, with 5 nM being applied for 3D7, and 10 nM for V1/S and CAM. Parasites recovered after transfection were cloned by limiting dilution (50) and genotyped prior to confirming barcode integration through sequencing.

### Parasite growth assay.

To examine parasite growth rate, parasitemia of individual lines was measured by flow cytometry (staining with 1xSYBR green and 150 nM mitotracker deep red) and seeded at 0.5% starting parasitemia at 3% hematocrit. Three biological replicates were followed for 10 days, with daily measurements of parasitemia performed by flow cytometry. Cultures were adjusted to maintain parasitemia below 5%, and cumulative parasitemia calculated by factoring in all adjustments to culture volume. For each strain and replicate, the relative growth rate was calculated by normalizing their cumulative parasitemia at each time point to the average cumulative parasitemia value of the three 3D7 replicates at each time point. The relative growth rate was then averaged across all time points for each replicate.

### Competitive growth assays and barcode sequencing.

Uniquely barcoded clones were expanded and independently synchronized by a Percoll gradient to enrich for late segmenting schizonts, followed by sorbitol synchronization (47). Prior to the start of the assay, parasitemia was accurately measured by using flow cytometry and mixtures were made at equal starting parasitemia on day zero, aiming for a final parasitemia of 1% and equal representation of each strain. Cultures were diluted to maintain parasitemia between 1 and 5% (measured by microscopy) throughout the time course, which would define the day of collection. Assays were performed for 6–18 days, which based on previous work is sufficient to reveal growth differences with isogenic lines bearing drug resistance mutations (11). With the exception of the DHA-based assays, continuous drug pressure was applied throughout the assay across the following concentration ranges: i) chloroquine (20, 50, 60, 100, 500 nM), ii) mefloquine (10, 20, 50, 500 nM), and iii) piperaquine (10, 30, 50, 100, 500 nM). For the DHA-based assays, we performed a standard competition assay as above where constant pressure (10 nM) was applied over 18 days, feeding every other day from a premade stock. We also performed a parallel assay (“pulsed”) in which 6 h pulses of 10 nM DHA were applied every 24 h without regular synchronization or consideration of parasite stage, mimicking the short half-life of DHA. In addition to this, a modified RSA was used in which a lower concentration of DHA (250 nM vs 700 nM) allowed for an increased survival and easier readout for the resistant strain. As in the standard RSA, 0–3 h rings were pulsed for 6 h and the output of the pulse vs the DMSO control was measured with BarSeq. Assays were conducted in duplicate or triplicate, each in 10 mL culture volumes, and at different time points cells were collected and lysed with 0.05% saponin for genomic DNA isolation.

Genomic DNA was extracted using a Qiagen blood and tissue kit. 50 ng of extracted genomic DNA was used as a template for a nested PCR using KAPA HiFi 2X polymerase master mix. A first PCR was performed to amplify from outside the homology regions of *Pfrh3* to avoid amplification from stably maintained episomes, using primers p191 and p194, followed by a nested 2-step PCR using p1356 and p1357 containing adapters for Illumina sequencing (PCR1). See Table S4 in the supplemental material for primer sequences. Following adapter ligation, 5 μL of PCR1 were used with paired-end index primers (Nextera XT) for PCR2. PCR products were purified from this last reaction only, using a Macherey-Nagel PCR purification kit and eluted DNA was quantified using Qubit broad sensitivity kit, multiplexed and diluted to a final concentration of 4 nM. Samples were loaded onto an Illumina MiSeq sequencer, using a MiSeq reagent kit v2 (300 cycle). They were loaded at a low cluster density (<400k cluster density), and 50% of PhiX was spiked in, as described in Gomes et al. (2015) for low complexity libraries (52).

### Barcode counting and analysis.

Barcode counting was performed as in Gomes et al. (52). The output Illumina fastq reads from the MiSeq were separated by index tags representing the different time points, and then analyzed with a script that identified correct flanking sequences and exact matches of unique barcodes between these regions were counted. Using these barcode counts, the relative proportion of each strain and replicate in each sample was quantified. To calculate a measure of relative growth, the proportions at each time point were normalized by dividing by the corresponding barcode’s proportion at the start of the competition assay, and log-2-transformed. This results in an initial value of 0 with subsequent positive or negative deviation implying an increase or decrease in proportion respectively. We also performed an analysis in which for each time point we calculated the log-2 ratio of the proportion at this time point from the proportion in the previous time point, which we represent in the boxplots. For this analysis, the last time point in all experiments was removed to avoid low read-counts from some strains (in particular V1/S) causing high variability in the ratio due to small fluctuations in counts. We then normalized these per-time point growth values to the growth of 3D7, to calculate a measure of relative fitness.

### Data availability.

All data necessary to perform the analysis of the barcode sequencing of parasite strains is available as part of the supplementary material (Tables S2 and 3 in the supplemental material).

10.1128/mbio.00937-22.5 TABLE S2 Raw data for competition assays (BarSeq). Download Table S2, XLSX file, 0.1 MB. Copyright © 2022 Carrasquilla et al. 2022 Carrasquilla et al. https://creativecommons.org/licenses/by/4.0/ This content is distributed under the terms of the Creative Commons Attribution 4.0 International license.
